# Supplementary material for: Ribosome-Profiling Reveals Restricted Post Transcriptional Expression of Antiviral Cytokines and Transcription Factors during SARS-CoV-2 Infection
Source: Int J Mol Sci. 2021 Mar 25;22(7):3392. doi: 10.3390/ijms22073392 (PMC8036502; doi:10.3390/ijms22073392)
Supplement: Supplementary file 1 [file ijms-22-03392-s001.zip › Supplementary Figures.pptx]

## Slide 1
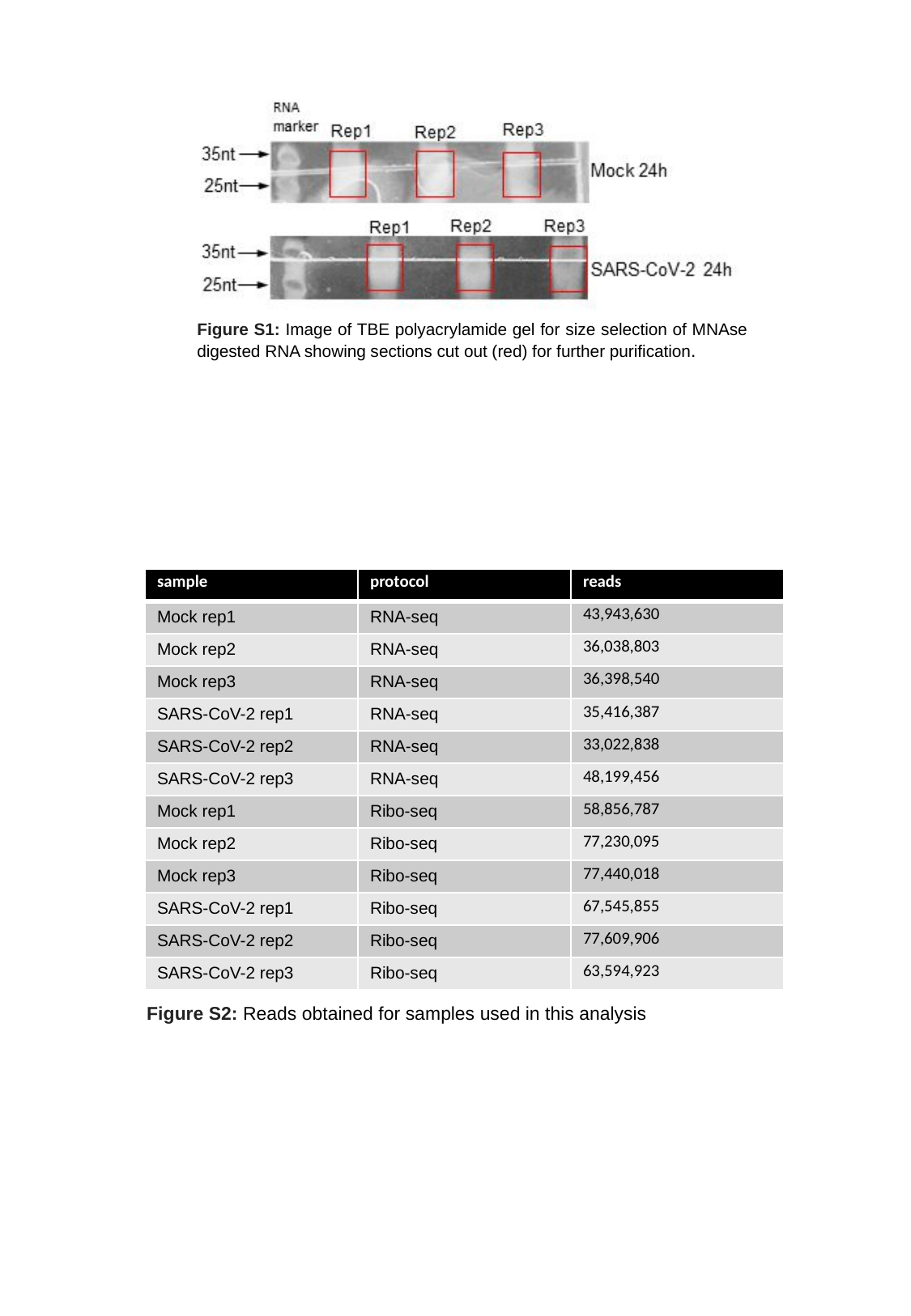

Figure S1: Image of TBE polyacrylamide gel for size selection of MNAse digested RNA showing sections cut out (red) for further purification.
| sample | protocol | reads |
| --- | --- | --- |
| Mock rep1 | RNA-seq | 43,943,630 |
| Mock rep2 | RNA-seq | 36,038,803 |
| Mock rep3 | RNA-seq | 36,398,540 |
| SARS-CoV-2 rep1 | RNA-seq | 35,416,387 |
| SARS-CoV-2 rep2 | RNA-seq | 33,022,838 |
| SARS-CoV-2 rep3 | RNA-seq | 48,199,456 |
| Mock rep1 | Ribo-seq | 58,856,787 |
| Mock rep2 | Ribo-seq | 77,230,095 |
| Mock rep3 | Ribo-seq | 77,440,018 |
| SARS-CoV-2 rep1 | Ribo-seq | 67,545,855 |
| SARS-CoV-2 rep2 | Ribo-seq | 77,609,906 |
| SARS-CoV-2 rep3 | Ribo-seq | 63,594,923 |
Figure S2: Reads obtained for samples used in this analysis

## Slide 2
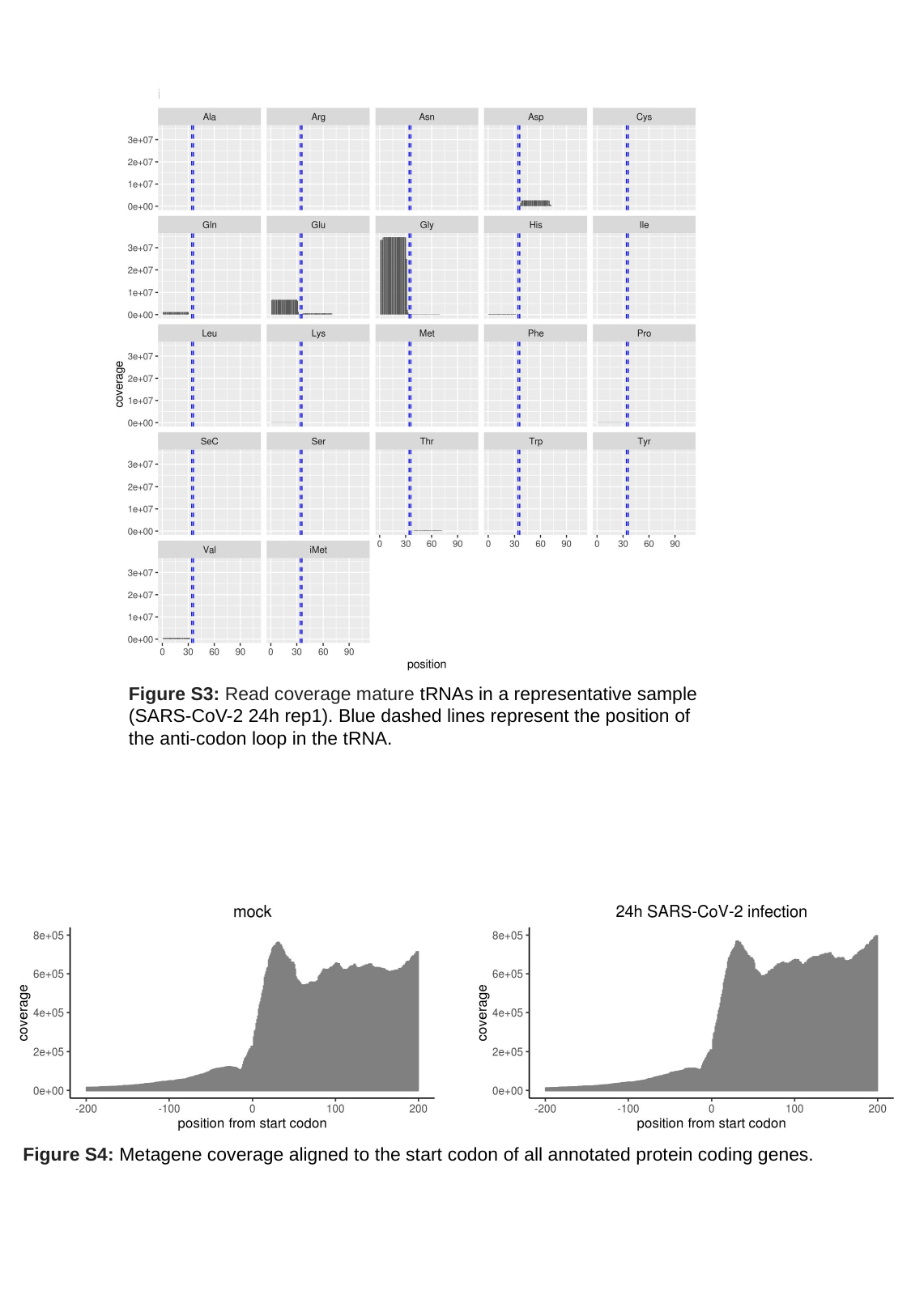

Figure S3: Read coverage mature tRNAs in a representative sample (SARS-CoV-2 24h rep1). Blue dashed lines represent the position of the anti-codon loop in the tRNA.
Figure S4: Metagene coverage aligned to the start codon of all annotated protein coding genes.
